# Functional Response and Intraspecific Competition in the Fall Armyworm, *Spodoptera frugiperda* (Lepidoptera: Noctuidae)

**DOI:** 10.3390/insects11110806

**Published:** 2020-11-16

**Authors:** Qilin Ren, Muhammad Haseeb, Jingyu Fan, Pengxiang Wu, Tianqi Tian, Runzhi Zhang

**Affiliations:** 1 Department of Entomology, Guizhou University, Guizhou 550025, China; qlren94@163.com; 2 Center for Biological Control, College of Agriculture and Food Sciences, Florida A&M University, Tallahassee, FL 32307-4100, USA; 3 Key Laboratory of Zoological Systematics and Evolution, Institute of Zoology, Chinese Academy of Sciences, Beijing 100101, China; jingyufan@hotmail.com (J.F.); wupengxiang@ioz.ac.cn (P.W.); tiantianqi15@mails.ucas.edu.cn (T.T.); 4 College of Life Sciences, University of Chinese Academy of Sciences, Beijing 100049, China

**Keywords:** FAW, cannibalism, insecticide application, functional response, intraspecific competition

## Abstract

**Simple Summary:**

The fall armyworm (FAW), *Spodoptera frugiperda* (J.E. Smith) (Lepidoptera: Noctuidae) is endemic to the Western Hemisphere, where it is particularly damaging to corn, and has become invasive to Africa and Asia in 2016, and 2018, respectively. In January 2019, the FAW was first found in southwest China, and by September 2019 it reached almost all southern Chinese provinces. Since China is the second largest corn producer in the world, it is necessary to study the feeding behavior of the FAW to provide a valuable reference for Chinese corn production and food security. In this study, the functional response and intraspecific competition of the FAW larvae were evaluated under the laboratory conditions. Our study indicates that fifth and sixth instar larvae can cause major host plant damage. Therefore, these should be the focus of any pest management strategy. Intraspecific competition, especially cannibalism impacts the feeding patterns of the FAW larvae and thus needs close attention. Studying the functional response and intraspecific competition could bring tremendous insights into the FAW-host and the FAW-FAW interactions.

**Abstract:**

Functional responses of the fall armyworm (FAW) larvae at each stage, and their intraspecific competition associated with cannibalism, provide insights into developing pest management strategies for the FAW. To help use insecticides more sparingly, the functional response and intraspecific competition of the FAW larvae were evaluated under the laboratory conditions. The results showed that all stages of the FAW larvae displayed a type II functional response to diet. Based on Holling’s disc equation, the search rate (*a*) and handling time (*T*_h_) of sixth instar larvae (*a* = 0.493; *T*_h_ = 0.37 min) were the highest, and the shortest of all larval stages, respectively. Intraspecific competition curves fitted the data for fourth to sixth larval stages of the FAW, and the coefficient of intraspecific competition (*m*) assessed by the intraspecific competition equation were highest for fifth instar larvae (*m* = 0.48). The present study indicates that 5th and 6th instar larvae can cause the most plant damage (accounted for 88.9% of larval consumption), and these stages should be the focus of any pest management strategy. Intraspecific competition, especially cannibalism, impacts the feeding patterns of the FAW larvae and needs close attention. Understanding the functional response and intraspecific competition of the FAW larvae contributes greatly to practical applications of insecticides, increasing the effectiveness of chemical sprays and decreasing ecological damage.

## 1. Introduction

The Fall armyworm (FAW), *Spodoptera frugiperda* (J.E. Smith) (Lepidoptera: Noctuidae) is a serious pest native to the Americas [1]. The FAW has a wide host range including 353 plant species within 76 families, many of them are economically important crops such as rice, corn, sugarcane and sorghum [2,3]. Over the past several years, the FAW has caused considerable economic damage to plant growth across the world [4]. The FAW is one of the most serious pests of corn, causing up to 73% loss in corn yields [5,6,7]. In corn, the FAW larvae feed on young leaf whorls, ears and tassels, and mature larvae can cut through the base of the seedlings, killing the whole plant [8]. Controlling the FAW is difficult as pesticides cannot penetrate the dense canopy of corn, hence exacerbating the challenge of pesticide use. The FAW lives year-round in the tropical and sub-tropical regions of the Americas, and undergoes seasonal migrations as far as north to temperate North America [9,10,11]. Since 2016, the FAW has begun to spread at a phenomenal rate in West Africa. Within two years of arrival in West Africa, the pest has reached 28 sub-Saharan African countries and is a serious concern for small scale and commercial growers [12,13]. Then, the moth invaded the eastward direction and successively reached India, Myanmar and Thailand in 2018 [14]. In January 2019, the FAW was first found in Yunnan province, southwest China, and by September 2019 it has reached almost all southern Chinese provinces [15]. China is the second largest corn producer in the world, corn is grown in almost all the provinces in China [1]. Studying the feeding behavior of the FAW larvae will provide valuable information for Chinese corn production and food security.

Cannibalism is a widespread natural behavior within many insect species, causing a profound impact on population dynamics [16,17]. Cannibals may confer a direct fitness benefit by increased survival, developmental rate, and fecundity, or it may provide indirect benefits by removing potential competitors and intraspecific predators [18,19,20]. Cannibalism is common in the larvae of Lepidoptera, although it can vary markedly between species [21,22,23]. From a pest management perspective, cannibals risk injury or death from defensive responses of conspecific and may cause an extra cost by consuming conspecifics infected with pathogens or parasites [24]. Intraspecific competition may cause a reduction in inclusive fitness via cannibalism [25]. Therefore, it is necessary for a pest management program to take advantage of intraspecific competition among the FAW larvae. Our preliminary observations indicate that feeding efficiency (host plant consumption rate) is greatly reduced when the FAW larvae density increased in an infested crop area. Due to competition for resources (food and space), intraspecific competition affects the feeding efficiency of the FAW larvae. Intraspecific competition is a density-dependent process, including indirect and direct interactions between conspecifics [26]. As an extreme case of intraspecific competition, cannibalism is the direct interaction within the species. There are several models for quantifying intraspecific competition using phenomenological [27] or mechanistic [28,29,30] approaches, suggesting that the feeding behavior depends on not only food quantity but also the FAW density. Therefore, it is essential to take into consideration the effect of intraspecific competition in practical applications. In addition, studying the functional response and intraspecific competition could bring tremendous insights into the FAW-host and the FAW-FAW interactions, which provides a better strategy for the control of the FAW. Therefore, our goal in this study was to determine the functional response and intraspecific competition of the FAW larvae under laboratory conditions, in order to provide an important reference for insecticide applications in open fields.

## 2. Materials and Methods

### 2.1. Insects

The FAW larvae, used in the experiment, were obtained from a greenhouse at the experimental farm of the Institute of Zoology, Chinese Academy of Sciences, located in the plant protection and plant inspection station of Huize, Yunnan province, China. Approximately 20 larvae were reared in a plastic container (16 × 22 × 8 cm high) on a modified artificial diet based on wheat germ, soybean bran, casein, and brewer’s yeast [31]. Larvae were reared at L:D 14:10, 26 ± 2 °C and 50 ± 10% RH. Prior to the studies, the FAW larvae used in experiments were transferred to Petri dishes (9 cm diameter), the artificial diet was supplied at 12 h intervals in order to guarantee an abundance of the FAW for experimentation. For the experiment, individuals of the FAW larvae were selected for study within 12 h of molting. They were starved for 24 h, and then placed in Petri dishes containing artificial diet. The dishes were kept at L:D 14:10, 26 ± 2 °C and 50 ± 10% RH.

### 2.2. Functional Response

To determine the functional response of the FAW larvae at various stages (1st, 2nd, 3rd, 4th, 5th and 6th instars), they were each provided with 1000 mg, 1500 mg, 2000 mg, 2500 mg or 3000 mg of artificial diet, respectively. The consumption was determined after 24 h by recording the weight of the rest of diet using an analytical balance. During the trials, the 1000 mg, 1500 mg, 2000 mg, 2500 mg or 3000 mg of artificial diet without any treatment were provided to correct the changed weight due to desiccation. Each treatment was replicated 10 times.

### 2.3. Intraspecific Competition

To evaluate the effects of intraspecific competition on the feeding behavior of the FAW larvae, all larval instars (1st, 2nd, 3rd, 4th, 5th and 6th instars) were studied. Newly emerged larvae are greenish with a black head. The head turning orangish in the second instar. In the second, and particularly the third instar, the dorsal surface of the body becomes brownish, and lateral white lines begin to form. In the fourth to the sixth instars the head is reddish brown, mottled with white, and the brownish body which bears white subdorsal and lateral lines.

The quantities of diet provided were 1000 mg, 2000 mg, 3000 mg, 4000 mg and 5000 mg, respectively, for 1, 2, 3, 4, and 5 FAW larvae in Petri dishes. The ratio of diet quantity to the FAW was kept at 1000 mg for each number of larvae. The increase in the number of larvae in each Petri dish would result in increasing competition between the FAW larvae for space. The coefficients of intraspecific competition were calculated based on the weight of the rest of diet after 24 h. Moreover, the 1000 mg, 2000 mg, 3000 mg, 4000 mg and 5000 mg of food without treatment were used to accurately determine the mass of diet. Each treatment was replicated 10 times.

### 2.4. Statistical Analysis

#### 2.4.1. Functional Response

The functional responses were determined using a two-stage analysis [32]. In the first step, cubic logistic regression analysis of the proportion of consumption as a function of provided diet quantity was carried out to determine the shape (type II or type III) of the functional responses,

(1) $$\frac{N_{a}}{N_{0}}=\frac{\exp(P_{0}+P_{1}N_{0}+P_{2}N_{0}^{2}+P_{3}N_{0}^{3})}{1+\exp(P_{0}+P_{1}N_{0}+P_{2}N_{0}^{2}+P_{3}N_{0}^{3})}$$

where *N_a_* is the quantity of diet consumed (mg) and *N*_0_ is the quantity of diet provided (mg). *P*_0_, *P*_1_, *P*_2_ and *P*_3_ are the intercept, linear, quadratic, and cubic coefficients, respectively. Negative or positive linear coefficients (*P*_1_) indicate a type II or type III functional response, respectively [32]. If a cubic equation non-significantly yields coefficients, it is desirable to reduce the model by eliminating the quadratic and cubic coefficients from Equation (1) and to retest the other parameters [32]. Given that logistic regression analysis indicated that our data fit type II in each case, further analyses were restricted to type II functional response. The Holling’s disc (Equation (2)) [33] was used to model the relationship between the quantity of diet consumed (*N_a_*) and the quantity of diet provided (*N*_0_),

(2) $$N_{a}=\frac{aTN_{0}}{1+aT_{h}N_{0}}$$

where *N_a_* and *N*_0_ are described in Equation (1), *T* is the total time, which in this case is 24 h, *a* is the searching rate and *T*_h_ is the handling time of every milligram of food. A nonlinear regression procedure (NLR) based on the Levenberg-Marquardt method was used to estimate the parameters *a* and *T*_h_. The starting values of *a* and *T*_h_ required by the NLR procedure were found using the linear regression of 1/*N*_a_ against 1/*N*_0_. The resultant y-intercept is the initial estimate of *T*_h_ and the reciprocal of the regression coefficient is an estimate of *a* [34,35].

#### 2.4.2. Intraspecific Competition

The experiment was performed to calculate the coefficients of intraspecific competition between the FAW larvae. Nonlinear regression analysis was used to estimate parameters of intraspecific competition model by fitting Equation (3) [27],

(3) $$E=QP^{-m}$$

where *E* is the mean consumption by the FAW larvae (mg), *P* is the number of the FAW larvae, *m* is the coefficient of intraspecific competition and *Q* is the theoretical maximum consumption (mg). The values of *Q* and *m* were obtained by the power-exponential regressing *E* and *P*.

Descriptive statistics are the mean values and standard errors of the mean. Differences between the weight loss due to desiccation and 0 were examined using one sample *t*-test. Statistical analyses were carried out using SPSS 20.0 software (IBM, Armonk, NY, USA). Regression analyses were done using SigmaPlot 12.0 software (Systat Software Inc., San Jose, CA, USA). *p* values < 0.05 were considered significant.

## 3. Results

### 3.1. Functional Response

Regardless of diet quantity provided, the weight losses due to desiccation were not significantly different from 0 (*t*-test, *p* > 0.05) during experiments. Therefore, the drying out of the diet were negligible during the experimental period. Parameter estimates from the logistic model (Equation (1) of the proportion of consumption by the FAW larvae over a 24 h period versus diet quantity are presented in Table 1. Estimates of the linear parameter *P*_1_ were significantly negative for all developmental stages (Table 1). Therefore, the logistic model analysis of all the developmental stages indicated a type II functional response to diet.

**Figure 1:**
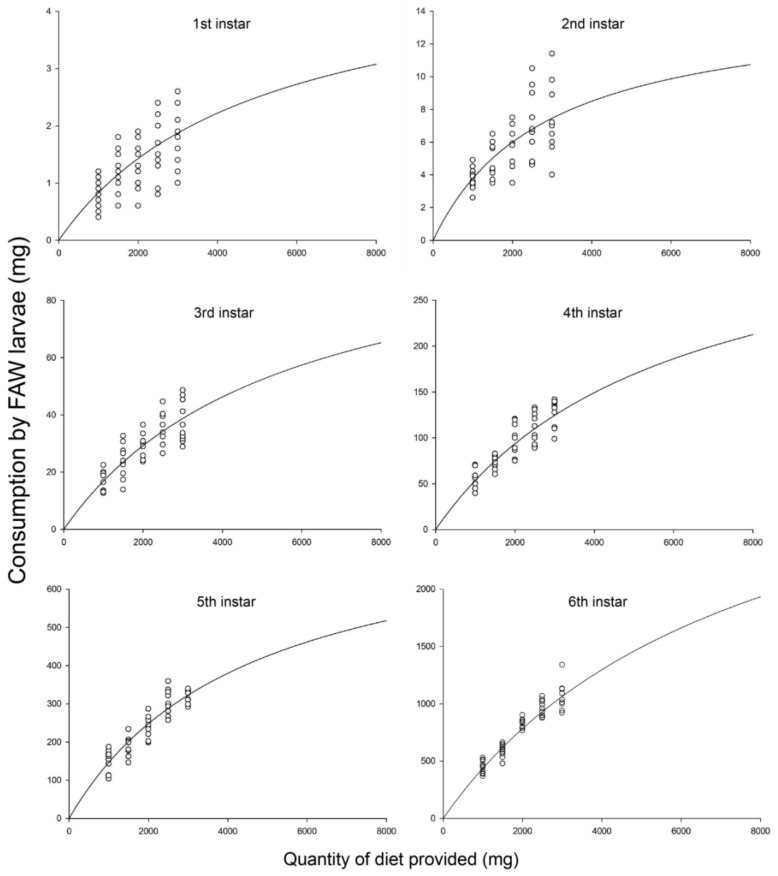
Functional responses at each larval stage of the FAW. Solid lines show the functional response curves of the FAW larvae on diet by fitting a Holling’s disc equation (Equation (2)). Circles show the consumption by the FAW when various quantities of diet were provided.

The functional response data for the FAW larvae consumption over a 24 h period were a good fit to Holling’s disc model (Equation (2)) (Table 2), confirming a type II functional response for all larval stages. The consumption increased significantly as the diet quantity provided increased, and the consumption ability of the FAW increased progressively with growth stage (Figure 1). Coefficients of searching rate (*a*) and handling time (*T*_h_) numerically indicated this relationship, which had asymptotic 95% confidence intervals that did not include 0 for all developmental stages. The searching rate of sixth instar larvae (*a* = 0.493) was the highest of all the larval stages, followed by the fifth (0.179), fourth (0.063), 3rd (0.020), second (0.005) and first instar (0.001) larvae. Moreover, the time sixth larvae took to handle every milligram of food (*T*_h_ = 0.37 min) was shortest, followed by the fifth (1.81 min), fourth (3.80 min), third (12.88 min), second (100.98 min) and first instar (296.47 min) larvae (Table 2).

### 3.2. Intraspecific Competition

**Figure 2:**
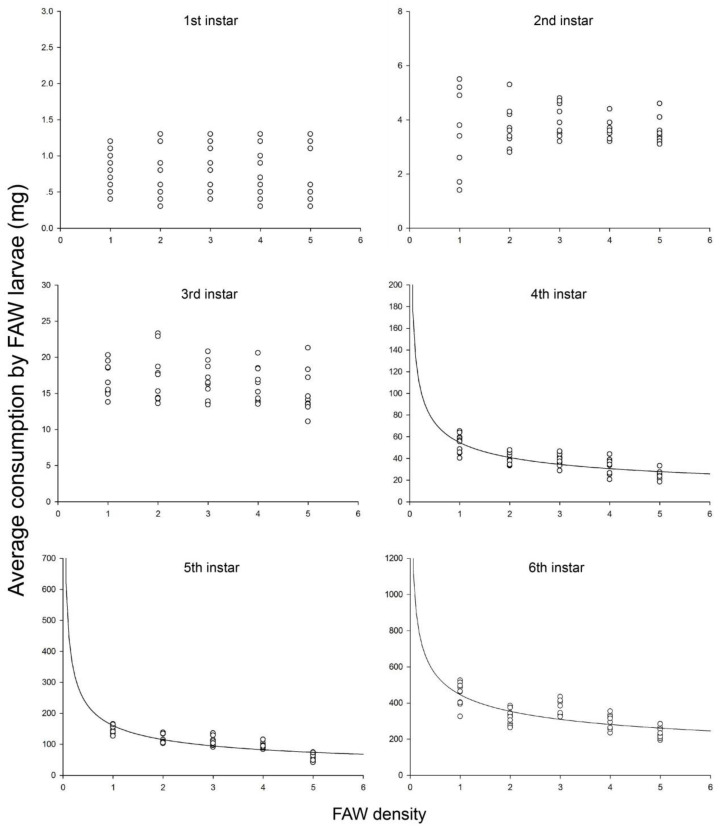
Intraspecific competition among the FAW larvae at each stage. Each data point represents the mean consumption by the FAW larvae at each FAW density. Curves were fitted using the intraspecific competition equation (Equation (3)).

Consumptions by the first (*F*_4,45_ = 0.061, *p* = 0.993), second (*F*_4,45_ = 0.374, *p* = 0.826) and third (*F*_4,45_ = 0.899, *p* = 0.472) instar larvae did not undergo a major change among different FAW densities (Figure 2). The mean consumptions at various FAW densities were 0.77 ± 0.05 mg (first instar larva), 3.72 ± 0.12 mg (second instar larva) and 16.35 ± 0.39 mg (third instar larva).

For the fourth, fifth and sixth instar larvae, when the ratio of diet quantity to the FAW was kept at 1000 mg, the total consumption per Petri dish gradually increased as the quantity of diet and the number of the FAW increased. However, the mean consumption by the FAW larvae at various stages decreased with increasing the FAW density due to intraspecific competition with limited space (Figure 2). Overall, the mean consumption by the 4th, 5th and 6th instar larvae at various FAW densities were 216.5 (diet/FAW = 1000 mg/1), 159.0 (2000 mg/2), 169.3 (3000 mg/3), 139.6 (4000 mg/4) and 107.6 (5000 mg/5). Intraspecific competition curves based on equation 3 fitted the data for all the three larval instars. At the three larval stages, the mean consumption at the different FAW densities fitted the intraspecific competition equation well (Table 3). The theoretical maximum consumption (*Q*) and coefficients of intraspecific competition (*m*) of all developmental stages had asymptotic 95% confidence intervals that did not include 0. The order of theoretical maximum consumption (*Q*) was highest for the sixth instar larvae (445.8 mg), followed by the fifth (160.5 mg) and fourth (54.7 mg) instar larvae. The order of coefficients of intraspecific competition (*m*) was highest for the fifth instar larvae (0.48), followed by the fourth (0.42) and sixth (0.33) instar larvae (Table 3).

## 4. Discussion

### 4.1. Functional Response

Our study indicated that all the larval stages of the FAW exhibited a type II functional response when performing feeding behaviors. Type II functional response curves have been reported in many studies on insect predation via the two-stage analysis [36,37,38,39]. Holling’s disc equation is the most common model used to analyse type II functional responses [40,41]. Our study also indicated the consumption by the FAW larvae increased but the consumption rates diminished with the increasing quantity of diet provided. This can be predicted by Holling’s disc model. Similar studies on other species of herbivore have also indicated the suitability of Holling’s disc model for describing the feeding behaviors [42,43,44].

We found that the feeding efficiencies of first and second instar larvae were low as they were small and moved slowly. Compared to the young instar larvae, third and fourth instar larvae consumed relatively at the higher rates. The fifth and sixth instar larvae accounted for 20.1%, and 68.8% of the total consumption, respectively. This indicates that mature larvae can play a major role for the FAW infestations and outbreaks. The basic premise of pest control is to control the pest before it becomes injurious and the first–third instar larvae should be the focus of the pest management practices. In fact, insecticides were often used a little late after the FAW infestations were observed. From a maize production perspective, fifth and sixth instar larvae are the major source of economic damage. Thus, it is necessary for pest management program to control the FAW more efficiently. Since the duration from egg to fourth instar larva is 7.48–11.12 days [45], there is at least a 7-d efficient window of insecticide applications to the FAW infestations on plants. Since the last two instars of the FAW consumed the largest amount of food and were the most injurious to crop, management practices should aim at controlling the young larvae before they reach the late instars and potentially cause heavy damage. Moreover, sixth instar larvae of the FAW were more voracious than the other larval stages, most likely due to higher nutritional requirements for development and pupation.

### 4.2. Intraspecific Competition

For the fourth, fifth, and sixth instar larvae, although the ratio of diet quantity to the FAW was kept constant at 1000 mg, the feeding efficiency decreased progressively along with the increased in the FAW densities in a Petri dish. Therefore, the mean consumption by the FAW larvae was adversely affected at the high densities due to an increased probability of intraspecific competition, especially the cannibalism. Many previous studies have showed intraspecific competition increased with the increasing population density of herbivores [46,47]. The intraspecific competition model (Equation (3)) is generally used to estimate the intraspecific competition [48,49]. The intraspecific competition equation for the FAW larvae yielded values for the parameters *Q* (theoretical maximum consumption) and *m* (coefficient of intraspecific competition).

Although when food is sufficient, the cannibalism can be frequently observed in a laboratory reared population of the FAW larvae. The high mortality of older larvae (fourth, fifth and sixth instar) was also observed during the experiments of intraspecific competition. Cannibalism between same-age larvae reared together is also possible, it mainly depends on feeding regime and rearing density. Cannibalism is prevalent among wild populations on the corn [50,51]. The FAW larvae feed primarily within the wrapped leaves of the whorl in young plants, so the frequent contact results in intraspecific competition [52,53]. Hence, the Petri dishes were provided to imitate the feeding regime with limited space. In the field, several younger FAW larvae were frequently observed feeding in the same whorl, whereas mature larvae almost never cohabit [54,55]. It is possible that nutritional benefits related to cannibalism are most important in the latter stages of the larval development. The incidence of cannibalism on the same-age individuals varies throughout larval development [56]. The cannibalism mainly occurred in mature larvae (4th–6th instars) of the FAW. Besides the FAW, cannibalism is also reported in other species within the Lepidoptera [19,22,57,58].

The quantity of food has an inverse relationship with the incidence of cannibalism [16]. The degree of crowding is also important, the incidence of cannibalism is greater at high densities [51]. When the degree of crowding is gradually increased, the frequency of cannibalism was affected by the age of individuals involved. Older individuals were more voracious cannibals than younger individuals in many species [59]. Thus, fifth and sixth instar larvae experienced a higher incidence of cannibalism. They had a higher coefficient of intraspecific competition than fourth instar larvae. Although, the maximum consumption of sixth instar larvae was highest in the larval stages, they became less active due to getting ready for the pupation. Occasionally, the cannibalism of the 6th instar larvae was not observed in individuals as early as 2-day-old. Hence, fifth instar larvae had a higher coefficient of intraspecific competition than the larvae at other stages. Intraspecific competition affects the feeding efficiency of the FAW larvae, that deserves the attention.

### 4.3. Remaining Questions and Future Perspectives

Feeding abilities, including searching rate and handling time are critical for estimating the economic damage of the FAW in maize production. Dynamics between the FAW and host plants can be evaluated using mathematical models [60]. Therefore, the dynamics can be established by using functional response curves, to determine the extent to which the feeding ability of the FAW depends on the food quantity of host plants in natural habitats. However, field studies are needed to validate the dynamics, because quantitative models based on laboratory studies appear to have limited value for determining feeding abilities under the field conditions [61,62]. For example, a series of studies on functional responses of *Podisus maculiventris* records differed under the laboratory and field studies [63,64,65]. This is most likely as the searching rates recorded in the laboratory and field conditions differed [66]. Spatial complexity, critical in the natural environment are not replicated in the simple laboratory conditions [67]. Laboratory studies provide parametric analysis of density-dependent intraspecific competition models, but these are carried out using only at a non-spatial scale. Thus, it is critical to measure intraspecific competition in spatially complex situations in the future studies as it is closer to natural conditions [68]. Intraspecific competition may disrupt the feeding capacities quantified by the functional responses. As such, understanding not only the FAW-host but also FAW-FAW interactions are vital in controlling the FAW. The functional response and intraspecific competition models describe the feeding behavior of the FAW accurately and also indicate cannibalism can impact its feeding efficiency. Thus, a comprehensive analysis of functional responses and intraspecific competition will result in a further improvement in our understanding of FAW-FAW-host interactions from the viewpoint of controlling the FAW. Moreover, natural stochasticity also needs to be taken into consideration in further field experiments on intraspecific competition among insects [69]. Plant characteristics also need to be considered because of their impact on the feeding efficiency of insects [70]. Further field investigations related to the FAW-host dynamics are critical for effective pest management, which potentially reduce chemical pesticide usage, preserving human health and environment [71,72,73].

## 5. Conclusions

All stages of the FAW larvae displayed a type II functional response to diet. Based on Holling’s disc equation, the search rate (*a*) and handling time (*T*_h_) of sixth instar larvae (*a* = 0.493; *T*_h_ = 0.37 min) were the highest, and the shortest at larval stages, respectively. Intraspecific competition curves fitted the data for fourth, fifth and sixth larval stages of the FAW, and the coefficient of intraspecific competition (*m*) assessed. The intraspecific competition equation was highest for the fifth instar larvae (*m* = 0.48). The present study determined that fifth and sixth instar larvae can cause a major economic damage (accounted for 88.9% of larval consumption). The basic premise of pest control is to control the pest before it becomes injurious and the first–third instar larvae should be the focus of the pest management practices. Therefore, management practices should aim at controlling the young larvae before they reach the late instars and potentially cause heavy damage. Intraspecific competition, especially cannibalism, impacts the feeding patterns of the FAW larvae and thus needs a close attention. Understanding the functional response and intraspecific competition for the FAW larvae contributes greatly to the practical application of insecticides, increasing the effectiveness of chemical sprays and decreasing ecological damage.

## Figures and Tables

**Table 1:** Maximum likelihood estimates (±SE) for parameters of the logistic model fit to proportion of prey consumed versus initial prey density.

Larval Instar	*P* ^0^	*P* ^1^	*P* ^2^	*P* ^3^
1st	−2.12654 **	−0.04317 **	0.00022 *	−4.67219
(SE)	0.122	0.006	0.00000042	0.21
2nd	−1.4321 **	−0.00531 *	0.000023	−0.00000012
(SE)	0.128	0.009	0.0000002	0.000000031
3rd	−0.20345	−0.00711 *	0.000034	−0.000000082
(SE)	0.091	0.006	0.0000084	0.000000007
4th	2.14786 *	−0.004253 *	−0.000027	0.000000077
(SE)	0.523	0.037	0.0000063	0.00000003
5th	2.53204 **	−0.054678 **	0.000375	−0.00000063
(SE)	0.311	0.024	0.0000694	0.00000011
6th	2.24428 **	−0.0132424 *	0.0000362	−0.00000003
(SE)	0.104	0.008	0.00000627	0.000000017

* Significant at *p* < 0.05; ** Significant at *p* < 0.01.

**Table 2:** Parameter estimates of the Holling’s disc equation (Equation (2) for the consumption by the FAW larvae.

Larval Instar	*R* ^2^	*F*_1,3_	*p*	Holling’s Disc Equation	*a*	*T_h_* (min)
1st	0.976	122.977	0.002	*N*_a_ = 0.0010*N*/(1 + 0.00020*N*)	0.001	296.47
2nd	0.991	330.687	<0.001	*N*_a_ = 0.0051*N*/(1 + 0.00035*N*)	0.005	100.98
3rd	0.996	709.639	<0.001	*N*_a_ = 0.0199*N*/(1 + 0.00018*N*)	0.02	12.88
4th	0.997	934.697	<0.001	*N*_a_ = 0.0627*N*/(1 + 0.00017*N*)	0.063	3.8
5th	0.986	208.166	0.001	*N*_a_ = 0.1786*N*/(1 + 0.00022*N*)	0.179	1.81
6th	0.983	171.244	0.001	*N*_a_ = 0.4932*N*/(1 + 0.00013*N*)	0.493	0.37

*R*^2^ is the coefficient of determination estimated by fitting Holling’s disc equations; *p* is the probability that Holling’s disc equation yields parameters; *a* is the searching rate; *T*_h_ is the handling time of every milligram of food.

**Table 3:** Parameter estimates of the intraspecific competition equation (Equation (3)) of consumption by the FAW larvae at various FAW densities.

Larval Instar	*R* ^2^	*F*_1,3_	*p*	Intraspecific Competition Equation	*Q* (mg)	*m*
4th	0.856	17.903	0.024	*E =* 54.6709*P*^−0.4185^	54.7	0.42
5th	0.804	12.289	0.039	*E =* 160.4898*P*^−0.4799^	160.5	0.48
6th	0.805	12.403	0.039	*E =* 445.7573*P*^−0.3325^	445.8	0.33

*R*^2^ is the coefficient of determination estimated by fitting intraspecific competition equations; *p* is the probability that intraspecific competition equation yields parameters; *Q* is the theoretical maximum consumption; *m* is the coefficient of intraspecific competition.
